# Data and programming code from the studies on the learning curve for radical prostatectomy

**DOI:** 10.1186/1756-0500-3-234

**Published:** 2010-09-02

**Authors:** Andrew J Vickers, Angel M Cronin

**Affiliations:** 1 Department of Epidemiology and Biostatistics, Memorial Sloan-Kettering Cancer Center, NY, USA

## Abstract

Our group analyzed a multi-institutional data set to address the question of how the outcomes of surgery for prostate cancer are affected by surgeon-specific factors. The cohort consists of 9076 patients treated by open radical prostatectomy at one of four US academic institutions 1987 - 2003. The primary analyses focused on 7765 patients without neoadjuvant therapy. The most well-known finding is that of a surgical "learning curve", with rates of prostate cancer cure strongly dependent on surgeon experience. In this "data note", we provide the raw data set, as well as well-annotated programming code for the main analyses. Data include markers of cancer severity (stage, grade and prostate-specific antigen level), cancer outcome, and surgeon variables such as training and experience.

## Introduction

We have long been proponents of scientific data sharing, having published articles advocating sharing of raw data[1], guidelines for preparing data sets[2,3] and empirical studies of authors' willingness to share data[4].

In some areas of science, such as microarray research, there are publicly available websites for depositing data (e.g. Gene Expression Omnibus or the Stanford Microarray Database). For many scientific fields, however, the primary means to share data is publication of supplementary files on the journal website. Yet journals vary in their policies as to supplemental files and, as such, an author might wish to make available the raw data from a study, but have no obvious venue for data publication. Moreover, investigators often conduct multiple analyses on a data set, publishing several different papers. This is a problem on the grounds that it is ideal for analytic code to be published alongside raw data sets. Even if a journal did agree to post supplemental raw data files to their website, it is unlikely that they would be sympathetic to publishing a comprehensive set of programming code encompassing analyses for papers previously published in other journals.

*BMC Research Notes *provides an excellent venue for posting data sets from studies published elsewhere in the literature. This is not only because *BMC *has liberal policies as to supplemental files, but because *Research Notes *is very flexible as to the form of scientific articles. This paper has been conceived as example of the sort of paper that might be published in *Research Notes *that serves primarily as a place holder for associated supplemental files, containing raw data and programming code.

## Research on the learning curve

In the early part of the 2000's, it became apparent that the results of cancer surgery could vary between surgeons, sometimes dramatically. Begg et al[5], for example, published data showing that many more surgeons that would be expected by chance had either very high or very low rates of surgical complications after radical prostatectomy. Colleagues of ours at Memorial Sloan-Kettering Cancer Center were interested in whether outcome variation might extend to cure rates. We collaborated with colleagues at the Cleveland Clinic and Wayne State in order to develop a large data set of patients undergoing radical prostatectomy. The data set included information as to patient's baseline risk (stage and grade of cancer, and level of prostate specific antigen), the surgeon who treated the patient, and the patient's outcome (date of relapse or last follow-up). On the basis of this data set, we have published 6 separate studies:

1. We demonstrated a "learning curve" for cancer control after radical prostatectomy. Patients treated by inexperienced surgeons were much more likely to recur than patients treated by more experience colleagues[6].

2. We found that the learning curve did not vary by either pre-operative risk[7] or pathologic stage[8]. Cure rates close to 100% in patients with organ-confined disease treated by the most experienced surgeons were taken to indicate that recurrences in such patients are primarily a function of surgical technique.

3. Recurrence rates vary between surgeons, even after adjusting for experience[9]. In other words, a patient may have a different chance of cure depending on which of two surgeons he sees, even if the two surgeons have conducted a similar number of previous radical prostatectomies.

4. There is a learning curve for surgical margins, although the poor concordance between a surgeon's margin and recurrence rates suggests that the former is not a good surrogate for the latter[10].

5. The surgical learning curve differs depending on fellowship training. Surgeons without fellowship training initially have similar recurrence results to their fellowship-trained colleagues but then fail to improve with experience. Conversely, the learning curve for surgical margin status did not differ by fellowship training. This suggests that there are different mechanisms of surgical learning for surgical margins and recurrence[11].

As our series of studies came to an end, our collaborators agreed to make the data set freely available for other investigators to use.

## Statement as to patient consent

Consent for publication of raw data was not obtained from participants. All data were obtained as part of routine clinical practice and were downloaded for research purposes under IRB waivers for retrospective data. Accordingly, consent for data publication was not obtained from patients before data were initially received. Obtaining consent retrospectively would be infeasible as there were over 9000 patients, some treated more than 20 years ago, and many of whom have died. Nonetheless, the dataset is fully anonymous in a manner that can easily be verified by any user of the dataset. Patients and surgeons are identified only by an anonymous code; there are no identifying data such as name, address or social security numbers; patient age is subject to random jitter; the age of patients who were unusually old or young at the time of surgery is modified to ceiling and floor values. As such, publication of the dataset clearly and obviously presents minimal risk to confidentiality of study participants.

## Supplemental files: data and statistical code

Given below is a list of files that we have uploaded to *BMC Research Notes *in order to make our data available. Following several prior recommendations [12], we have also uploaded statistical code to allow replication of our results. The code is saved as Stata "do" files, but these can be opened from within a text editor or word processing package such as Microsoft Word. The code has been well-annotated, we hope sufficiently so to allow non-Stata users to follow our logic. We created over 100 do files for the numerous papers associated with our learning curve studies. Publishing all of these do files would more likely lead to confusion than insight. As such, we selected a sub-set of representative analyses that we believe would allow any competent analyst to replicate our results. For example, we provide code for a sensitivity analysis that includes only surgeons whose career experience was at least 100 cases; this code is easily adapted for a sensitivity analysis that includes surgeons with career experience of 250 or more cases.

Moreover, some of the code was originally written in a highly modular fashion, with kernels of code referenced by numerous different do files, with extensive routines for printing out results in a readable form (e.g. rounding p values). Both features can make our programming difficult to follow. Accordingly, we simplified the code for this presentation, removing code associated with presentation, and duplicating code in different do files in some cases. We also wrote new code to deidentify the data set.

We estimate that the total time taken to prepare the data and code for publication was 8 hours. While far from trivial, this constitutes a small fraction of the effort spent on the data set over the past five years. Moreover, this estimate must be seen as higher than typical, given that the code involved covered so many different papers.

The data have been uploaded both in Stata format, and a raw format that can be read by most software (it can be opened directly in Microsoft Excel, for example). These two files are named "master learning curve data set deidentified" with ".dta" and ".raw" extension respectively. "Variable labels.pdf" describes each variable on the data set [see Additional files 1, 2 and 3]. A description of each do file is as follows:

**1. 01 deidentify data learning curve.do **[Additional file 4]

This do file takes the data set with identifying information and saves out a new data set without any identifying information. This includes removing patient and surgeon identifiers and replacing them with anonymous identifiers, removing dates, and ensuring that patient age cannot identify individuals. Before saving out the deidentified data set, a data set is saved with both the true and anonymous patient and surgeon identifiers; this data set is not published, but is kept with the primary investigators so that any data enquiries about individual patients can be addressed by the primary investigator.

**2. 02 primary analysis bcr learning curve.do **[Additional file 5]

This do file performs the primary analysis of the learning curve for biochemical recurrence[6]. This is an example of the code to produce a learning curve for a survival-time outcome. A multivariable analysis is performed to obtain the adjusted p-value for the association between surgeon experience and outcome; the adjusted 5-year predicted probability of freedom from biochemical recurrence is plotted against surgeon experience; and the central estimates for 10 and 250 prior cases are displayed.

**3. 03 bootstrap ci for difference in 10 vs 250 bcr learning curve.do **[Additional file 6]

This do file uses bootstrap resampling to construct a 95% confidence interval for the difference in adjusted 5-year probability of biochemical recurrence for a patient treated by a surgeon with 10 vs 250 prior cases[6]. The output from the bootstrap resampling is saved as a Stata data set "output bootstrap ci for difference 10 vs 250 learning curve.dta". This is an example of code where bootstrap resampling is used to obtain confidence intervals for an estimate whose sampling distribution is unknown. The code could be modified easily for another estimate of interest, for example, the difference in adjusted probability of positive surgical margins for a patient treated by a surgeon with 10 vs 250 prior cases.

**4. 04 sensitivity analysis patients treated after 1995 bcr learning curve.do **[Additional file 7]

This do file performs the same analysis as done in "02 primary analysis bcr learning curve.do", except that the cohort is restricted to patients treated after 1995, when stage migration related to the advent of PSA screening appeared to be largely complete[6]. This is an example of the code where a specific group of patients is included, and another group excluded. This code could be modified easily to restrict the analysis to a different subgroup, for example, patients with low risk disease.

**5. 05 sensitivity analysis surgeons with at least 100 total cases bcr learning curve.do **[Additional file 8]

This do file performs the same analysis as done in "02 primary analysis bcr learning curve.do", except that the cohort is restricted to surgeons who completed at least 100 total cases. This sensitivity analysis was performed to confirm that the relationship between surgeon and experience and outcome was not confounded by the ability of individual surgeons to attract patients (i.e., a less capable surgeon who was unable to establish a practice would therefore contribute to the beginning but not the end of the learning curve)[6]. This is an example of the code where only patients treated by a specific group of surgeons are included. This code could be modified easily to restrict the analysis to patients treated by a different group of surgeons, for example, surgeons who completed at least 250 total cases.

**6. 06 separately by postoperative risk bcr learning curve.do **[Additional file 9]

This do file performs the primary analysis of the learning curve for biochemical recurrence separately by pathologic stage[8]. This is an example of the code to produce a learning curve separately for different subgroups of patients. A multivariable analysis is performed to obtain the adjusted p-value for the association between surgeon experience and outcome separately for those with organ-confined and non-organ-confined disease; the adjusted 5-year predicted probability of freedom from biochemical recurrence is plotted against surgeon experience separately by pathologic stage; and the central estimates for 10 and 250 prior cases are displayed. This code could be modified to obtain separate learning curves for subgroups defined in other ways, for example, patients treated by fellowship vs. non-fellowship trained surgeons.

**7. 07 surgical margins learning curve.do **[Additional file 10]

This do file performs the primary analysis of the learning curve for surgical margins[10]. This is an example of the code to produce a learning curve for a binary outcome. A multivariable analysis is performed to obtain the adjusted p-value for the association between surgeon experience and outcome; the adjusted predicted probability of positive surgical margin is plotted against surgeon experience; and the central estimates for 10 and 250 prior cases are displayed. This code could be modified easily to restrict the analysis to a particular subgroup of patients.

**8. 08 heterogeneity in bcr by surgeon.do **[Additional file 11]

This do file performs a multivariable random-effects model to evaluate heterogeneity between surgeons in biochemical recurrence outcomes after adjustment for case-mix and surgeon experience. The random effects variance, 95% confidence interval, and p-value are displayed[9]. This is an example of the code to determine whether heterogeneity exists between surgeons, and could be modified easily for different types of outcomes (for example, a binary outcome such as positive surgical margins) or different subgroups of patients.

**9. 09 forest plot bcr by surgeon.do **[Additional file 12]

This do file obtains the adjusted 5-year predicted probability of freedom from biochemical recurrence for each surgeon; obtains a combined estimate across all surgeons using meta-analytic methods, and shows the probabilities and 95% confidence intervals for each surgeon as a forest plot[9]. This could be modified easily for different types of outcomes or different subgroups of patients.

## Conclusions

Publishing scientific papers on the web provides far greater flexibility of form and function than is possible with traditional publication in a paper journal. The medium allows the development of new kinds of scientific paper, such as a "Data Note", including data and statistical code for scientific projects involving several different research questions and multiple papers. This paper aims to provide an example of the form.

## Competing interests

The authors declare that they have no competing interests.

## Authors' contributions

AV conceived of the idea; AV and AC co-wrote the paper; AC amended the statistical code for publication. AV and AC read and approved the final version.

## Supplementary Material

Additional file 1 **master learning curve data set deidentified**. Tumor characteristics, surgeon, and outcome for all patients on the data set in Stata format. Click here for file

Additional file 2 **master learning curve data set deidentified**. Tumor characteristics, surgeon, and outcome for all patients on the data set in text format. Click here for file

Additional file 3 **Variable labels. Describes the variables in the data set**. Click here for file

Additional file 4 **deidentify data learning curve.do**. This is a Stata "do" file - statistical programming code - that takes the data set with identifying information and saves out a new data set without any identifying information. Click here for file

Additional file 5 **primary analysis bcr learning curve.do**. This is a Stata "do" file - statistical programming code - that performs the primary analysis of the learning curve for biochemical recurrence[6]. Click here for file

Additional file 6 **bootstrap ci for difference in 10 vs 250 bcr learning curve.do**. This is a Stata "do" file - statistical programming code - that uses bootstrap resampling to construct a 95% confidence interval for the difference in adjusted 5-year probability of biochemical recurrence for a patient treated by a surgeon with 10 vs 250 prior cases[6]. Click here for file

Additional file 7 **sensitivity analysis patients treated after 1995 bcr learning curve.do**. This is a Stata "do" file - statistical programming code - that performs the same analysis as done in "02 primary analysis bcr learning curve.do", except that the cohort is restricted to patients treated after 1995. Click here for file

Additional file 8 **sensitivity analysis surgeons with at least 100 total cases bcr learning curve.do**. This is a Stata "do" file - statistical programming code - that performs the same analysis as done in "02 primary analysis bcr learning curve.do", except that the cohort is restricted to surgeons who completed at least 100 total cases. Click here for file

Additional file 9 **separately by postoperative risk bcr learning curve.do**. This is a Stata "do" file - statistical programming code - that performs the primary analysis of the learning curve for biochemical recurrence separately by pathologic stage[8]. Click here for file

Additional file 10 **surgical margins learning curve.do**. This is a Stata "do" file - statistical programming code - that performs the primary analysis of the learning curve for surgical margins[10]. Click here for file

Additional file 11 **heterogeneity in bcr by surgeon.do**. This is a Stata "do" file - statistical programming code - that performs a multivariable random-effects model to evaluate heterogeneity between surgeons in biochemical recurrence outcomes after adjustment for case-mix and surgeon experience[9]. Click here for file

Additional file 12 **forest plot bcr by surgeon.do**. This is a Stata "do" file - statistical programming code - that obtains the adjusted 5-year predicted probability of freedom from biochemical recurrence for each surgeon; obtains a combined estimate across all surgeons using meta-analytic methods, and shows the probabilities and 95% confidence intervals for each surgeon as a forest plot[9]. Click here for file
