# Supplementary material for: Data and programming code from the studies on the learning curve for radical prostatectomy
Source: BMC Res Notes. 2010 Sep 2;3:234. doi: 10.1186/1756-0500-3-234 (PMC3161373; doi:10.1186/1756-0500-3-234)
Supplement: Additional file 3 — Variable labels. Describes the variables in the data set. [file 1756-0500-3-234-S3.PDF]

Variable name	Variable label
anonpatientid	anonymous patient identifier
nht	received neoadjuvant hormone therapy: 0=no, 1=yes
anonage	age at radical prostatectomy (years), with jitter of 1year randomly added
psa	pre-treatment PSA (ng/ml)
clinicalstage	clinical stage
biopsygleason	biopsy Gleason score
biopsygleasoncat	biopsy Gleason score in categories
surgyear	year of radical prostatectomy
anonsurgeonid	anonymous surgeon identifier
academic	faculty member of department of urology at current institution: 0=no, 1=yes
fellow	at least 1 year of urologic studies after residency: 0=no, 1=yes
nprior	no. prior RP's performed by a surgeon before the first case on data set
experience_noprior	no. prior RP's performed by surgeon; includes only patients on data set
experience	no. prior RP's performed by surgeon; includes patients on data set and prior
experiencecat	no. prior RP's in categories; includes patients on data set and prior
pathgleason	specimen Gleason score
pathgleasoncat	specimen Gleason score in categories
sm	surgical margin status: 0=negative, 1=positive
ece	extracapsular extension: 0=no, 1=yes
svi	seminal vesicle invasion: 0=no, 1=yes
lni	lymph nodes: 0=negative; 1=positive
nocd	non-organ confined disease: 0=no, 1=yes
bcr	experienced biochemical recurrence by last followup date: 0=no, 1=yes
dead	vital status at last followup date: 0=alive, 1=dead
dod	died from prostate cancer: 0=no, 1=yes
ttlastfollowbcr	months from radical prostatectomy to biochemical recurrence or last followup
